# Insufficient insulin administration to diabetic rats increases substrate utilization and maintains lactate production in the kidney

**DOI:** 10.14814/phy2.12233

**Published:** 2014-12-11

**Authors:** Christoffer Laustsen, Kasper Lipsø, Jakob Appel Østergaard, Rikke Nørregaard, Allan Flyvbjerg, Michael Pedersen, Fredrik Palm, Jan Henrik Ardenkjær‐Larsen

**Affiliations:** 1 Department of Clinical Medicine, MR Research Centre, Aarhus University, Aarhus, Denmark; 2 Danish Research Centre for Magnetic Resonance, Copenhagen University Hospital Hvidovre, Hvidovre, Denmark; 3 Department of Electrical Engineering, Technical University of Denmark, Kgs. Lyngby, Denmark; 4 Department of Endocrinology and Internal Medicine and Danish Diabetes Academy, Aarhus University Hospital, Aarhus, Denmark; 5 Department of Clinical Medicine, Faculty of Health, Aarhus University, Aarhus, Denmark; 6 Comparative Medicine Lab, Aarhus University, Aarhus, Denmark; 7 Department of Medical Cell Biology, Uppsala University, Uppsala, Sweden; 8 Division of Drug Research, Department of Medical and Health Sciences, Linköping University, Linköping, Sweden; 9 Center for Medical Image Science and Visualization, Linköping University, Linköping, Sweden; 10 GE Healthcare, Broendby, Denmark

**Keywords:** Hyperpolarization, kidney, magnetic resonance imaging, renal metabolism, type 1 diabetes

## Abstract

Good glycemic control is crucial to prevent the onset and progression of late diabetic complications, but insulin treatment often fails to achieve normalization of glycemic control to the level seen in healthy controls. In fact, recent experimental studies indicate that insufficient treatment with insulin, resulting in poor glycemic control, has an additional effect on progression of late diabetic complications, than poor glycemic control on its own. We therefore compared renal metabolic alterations during conditions of poor glycemic control with and without suboptimal insulin administration, which did not restore glycemic control, to streptozotocin (STZ)‐diabetic rats using noninvasive hyperpolarized ^13^C‐pyruvate magnetic resonance imaging (MRI) and blood oxygenation level–dependent (BOLD) ^1^H‐MRI to determine renal metabolic flux and oxygen availability, respectively. Suboptimal insulin administration increased pyruvate utilization and metabolic flux via both anaerobic and aerobic pathways in diabetic rats even though insulin did not affect kidney oxygen availability, HbA_1c_, or oxidative stress. These results imply direct effects of insulin in the regulation of cellular substrate utilization and metabolic fluxes during conditions of poor glycemic control. The study demonstrates that poor glycemic control in combination with suboptimal insulin administration accelerates metabolic alterations by increasing both anaerobic and aerobic metabolism resulting in increased utilization of energy substrates. The results demonstrate the importance of tight glycemic control in insulinopenic diabetes, and that insulin, when administered insufficiently, adds an additional burden on top of poor glycemic control.

## Abstract (precis)

This work describes the metabolic changes associated with insufficient insulin administration in the type 1 diabetic rat kidney, showing that poor glycemic control with insufficient insulin administration, has an cumulative effect on the development of late diabetic complications.

## Introduction

Good glycemic control delays the onset and progression of late diabetic complications (The Diabetes Control and Complications Trial Research Group 1993). However, even diabetic patients with appropriate glycemic control have a risk of complications such as development of diabetic nephropathy; a common complication affecting both type 1 and type 2 diabetic patients (Richard et al. 2011). The exact mechanism resulting in the onset and progression of diabetic nephropathy is not fully understood, but disturbed oxygen metabolism causing intrarenal tissue hypoxia is likely to contribute (Hansell et al. 2013). The diabetic kidney utilizes more oxygen and increasing amount of energy substrates (Körner et al. 1994; Hansell et al. 2013; Laustsen et al. 2013, 2014). The resulting substrate limitation may serve to control energy expenditure and oxygen utilization and prevent an even further aggravated hypoxia.

As there is no metabolic control of renal blood flow and since oxygen can be shunted from arterial to venous blood before reaching renal capillaries, increased kidney oxygen consumption results in renal tissue hypoxia (Levy and Imperial 1961; Leong et al. 2007). The balanced relationship between energy‐requiring active tubular transport of electrolytes and whole‐kidney oxygen consumption is significantly shifted in the diabetic kidney due to up‐regulation of basal metabolism, mitochondrial uncoupling (i.e., leak respiration), and reduced electrolyte transport efficiency (Hansell et al. 2013). Indeed, it has recently been demonstrated that renal tissue hypoxia per se, without confounding factors such as hyperglycemia, hypertension, or oxidative stress, is sufficient to induce both histological and functional changes characteristic of early stages of chronic kidney disease (Friederich‐Persson et al. 2013). Furthermore, we have recently reported that reduced oxygen availability in the diabetic kidney directly increases the anaerobic metabolic flux paradoxically without limiting aerobic metabolism (Laustsen et al. 2014).

Insulin facilitates cellular glucose uptake and aerobic metabolism, whereas the diabetic kidney already utilizes more energy substrates, which may serve to limit energy expenditure and oxygen utilization via the aerobic pathways and prevent an even further aggravated hypoxia.

Our hypothesis is that administration of insufficient insulin to normalize blood glucose levels accelerates the negative metabolic alterations occurring in the diabetic kidney. The hypothesis is tested in vivo by studying the metabolism of hyperpolarized [1‐^13^C]pyruvate by magnetic resonance imaging (MRI; Ardenkjaer‐Larsen et al. 2003, 2011; Kurhanewicz et al. 2011; Nelson et al. 2013; Rider and Tyler 2013; Keshari and Wilson 2014).

## Methods and Materials

### Animals

The study complied with the National Institute of Health (NIH) guidelines for use and care of laboratory animals and was approved by the Animal Experiments Inspectorate, under the Danish Veterinary and Food Administration, License no. 2010/561−1938. Twelve 8‐week‐old female Wistar rats were included in this study, and insulinopenic diabetes was induced in all animals by a tail vein injection of streptozotocin (STZ; 55 mg/kg body weight; Sigma–Aldrich, Brøndby, Denmark). Blood glucose was measured in tail capillary blood with a Contour blood glucose meter (Bayer Diabetes Care, Copenhagen, Denmark). Rats were considered diabetic when the blood glucose levels exceeded 15 mmol/L at 48 h after injection of STZ. Six randomly selected diabetic rats received two subcutaneous injections of insulin daily for 3 days (1 IE, morning, 3 IE evening; NPH insulin, Eli Lilly, Indianapolis, IN) before the MRI examination in order to induce a condition of suboptimal glycemic control in the presence of insulin (DM + I) as measured by blood glucose levels. Six diabetic rats remained untreated with insulin and served as controls (DM).

### In vivo hyperpolarized ^13^C metabolic MRI

Two‐to‐three weeks after induction of diabetes, tail vein catheterization was performed for administration of hyperpolarized [1‐^13^C]pyruvate. At the day of the MR examination, the animals were placed in the MRI system (4.7 T Direct Drive, VnmrJ 4.0 software; Agilent, Santa Clara, CA), equipped with a ^1^H/^13^C volume transmit/receive and 4‐channel surface array ^13^C receive coil setup (Rapid Biomedical, Würzburg, Germany). ^1^H BOLD MRI and ^13^C MR spectroscopic imaging were conducted during normoxic breathing conditions (0.8% isoflurane, 0.40 L/min oxygen, and 1.60 L/min nitrogen). Body temperature and respiration were monitored throughout the experimental session. The kidneys were localized by a standard gradient‐echo sequence, and a slice covering both kidneys was shimmed manually before the metabolic evaluation. An oxygenation‐dependent (R_2_*‐weighted) sequence was performed using an axial ^1^H‐based multi‐echo gradient‐echo sequence. The sequence parameters were as follows: matrix = 128 × 128, field of view (FOV) = 80 × 80 mm^2^, flip angle = 30°, repetition time (TR) = 300 msec, number of transients = 16, equidistant echo times from 2 to 16 msec, and three slices of 5‐mm thickness covering both kidneys. A scout ^1^H‐based axial gradient echo sequence (matrix = 128 × 128, FOV = 80 × 80 mm^2^, TR/TE = 130 msec/2.6 msec, flip angle = 20°, 4 averages and 24 slices of 2‐mm thickness, covering both kidneys) was acquired for anatomical overlay. A slice‐selective 2D ^13^C chemical‐shift imaging sequence was used for hyperpolarized [1‐^13^C]pyruvate imaging. The parameters were as follows: flip angle = 10°, a full centric circular k‐space trajectory with a matrix size of 16 × 16, TR/TE = 75 msec/0.65 msec, FOV = 60 × 60 mm^2^, spectral width of 4000 Hz with 256 complex points, and an axial slice thickness of 20 mm covering both kidneys.

### Hyperpolarization

A volume of 20 *μ*L [1‐^13^C]pyruvic acid (Sigma Aldrich, Brøndby, Denmark) containing 15 mmol/L trityl radical OX063 (Oxford Instruments, Oxford, U.K.) and 1.5 mmol/L Dotarem (Guerbet, Villepinte, France) was subjected to dynamic nuclear polarization using a HyperSense polarizer (Oxford Instruments Molecular Biotools, Oxford, U.K.). The solution was polarized for at least two time constants using 100 mW microwaves at 94.118 GHz. The hyperpolarized sample was dissolved in 4 mL of a dissolution medium (80 mmol/L TRIS, 100 mg/L EDTA, 50 mmol/L NaCl, 80 mmol/L NaOH) yielding an isotonic 80 mmol/L [1‐^13^C]pyruvate at physiological pH (Laustsen et al. 2013, 2014). The sample temperature after dissolution was 30–35°C. A volume of 1.0 mL was injected into the tail vein over 10 sec, followed by a flush with saline. The transfer time between dissolution and injection was 10 sec on average, and scanning was initiated 20 sec after start of injection.

### MRI analysis

Data analysis of BOLD MRI data was performed using the open source software Osirix (Rosset et al. 2004), generating R_2_* maps. CSI data were processed in Matlab (MathWorks, Natick, MA), and spatial dimensions were apodized with a hamming (*α *= 0.5435) function and zero‐filled to a 32 × 32 grid. The spectral dimension was centered on the pyruvate frequency; apodized with a zero shifted sine‐bell function and a 5 Hz exponential line broadening and processed as sum‐of‐squares of the four coil channels. The spectral analysis was performed as signal integrals over a 60 Hz region for each metabolite. The metabolite maps and anatomical images were imported to Osirix and ROI analysis performed. The metabolite ROI was normalized relative to either the pyruvate signal or the total hyperpolarized signal.

### Measurements of markers of oxidative stress damage in plasma and tissue

After the MRI session, a 5–7 mL blood sample was collected from the aortic bifurcation to EDTA coated tubes for determination of plasma electrolytes and urea (Roche Cobas 6000; Roche Diagnostic, Hvidovre, Denmark) and plasma osmolality (Osmomat 030; Gonotec, Berlin, Germany). Plasma and kidney cortex were collected and stored at −80°C for later analyzes of thiobarbituric acid reactive substances (TBARS) and protein carbonyls. For TBARS, plasma samples were diluted 1:5 with 50 mmol/L HCl, and 50 *μ*L diluted plasma was mixed with 150 *μ*L 0.67% thiobarbituric acid, vortexed and heated to 97°C for 60 min. After cooling on wet ice, 200 *μ*L methanol with 1 mmol/L NaOH (dilution 91:9) was added, samples vortexed and centrifuged at 850 *g* for 5 min at room temperature. The supernatant was analyzed for fluorescence (ex. 532 nm, em. 553 nm) and concentrations calculated using a standard curve of malondialdehyde (0.25–62.5 nmol/L). Tissue samples were homogenized in radioimmunoprecipitation assay (RIPA) buffer diluted 1:1 with 50 mmol/L HCl and analyzed as described above. Tissue concentrations were corrected for protein content in the homogenized sample. Plasma and tissue levels of protein carbonyls were analyzed using a commercially available kit (Cayman Chemicals, Ann Arbor, MI) according to manufacturer's instructions.

### Statistics

Normality was assessed with quintile plots. *P* < 0.05 (*) was considered statistically significant. Comparisons of animal and kidney information, metabolic response, and oxygen‐sensitive MRI measurements were analyzed with a two‐tailed Student's *t*‐test with equal variance. The statistical analysis was performed in PRISM 6 (GraphPad Software, La Jolla, CA).

## Results

### Effect of insulin on blood glucose, renal function, and oxidative stress in rats subjected to diabetes

All rats developed a robust hyperglycemia within 48 h after the STZ injection. Body weight, kidney weight, and long‐term glucose level (HbA_1c_) and general plasma parameters were similar in the two groups (Table 1). Markers of oxidative stress damage (TBARS and protein carbonyls) in kidney cortical tissue were similar in the two groups, respectively (*P* = 0.07, *P* = 0.37), whereas plasma TBARS was reduced in the diabetic animals receiving insulin (*P* = 0.04; Table 2).

**Table 1.** Changes in body weight, kidney weight, blood glucose, and the levels of plasma creatinine, urea, and electrolytes after insulin treatment.

	BW g	KW g	Blgl mmol/L	HbA_1c_ %	Crea *μ*mol/L	Urea mmol/L	Osm mOsm	Na^+^ mmol/L	K^+^ mmol/L
DM	216±14	1.1±0.1	28.6±1.6	11.0±1.1	23±4	12±2	347±34	149±15	4.5±1.0
DM + I	220±9	1.1±0.1	18.3±2.6*	10.3±0.9	20±4	9±3	306±62	138±33	4.2±0.9

BW, body weight; KW, kidney weight; Blgl, blood glucose, prior scan; HbA_1c_, hemoglobin A_1c_; Crea, plasma creatinine; Osm, plasma osmolality; Na^+^, plasma sodium, K^+^, plasma potassium.

Mean±SEM of *n* = 6/group.

* Denotes *P* < 0.05 versus untreated group.

**Table 2.** Markers of oxidative stress damage.

	TBARS		Protein carbonyls	
	Plasma nmol/L	Kidney cortex nmol/g	Plasma nmol/L	Kidney cortex *μ*mol/g
DM	8.84±0.13	1.70±0.14	17.0±2.6	2.8±1.5
DM + I	8.50±0.07*	1.30±0.14	16.9±2.7	2.2±0.3

TBARS, thiobarbituric acid reactive substances.

Mean±SEM of *n* = 6/group.

* Denotes *P* < 0.05 versus untreated group.

### Effect of insulin on metabolic activity in rats subjected to diabetes

**Figure 1.**
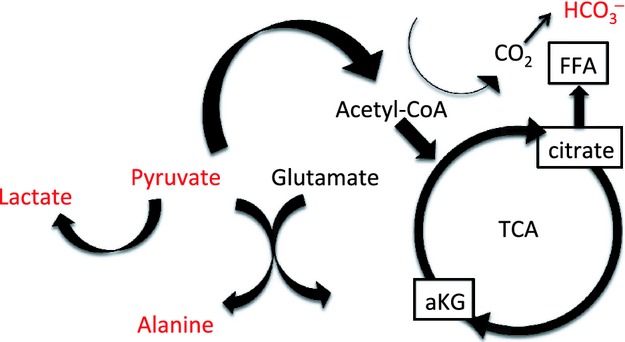
Diagram of the metabolic pathways observed in the hyperpolarized [1‐^13^C]pyruvate experiment.

**Figure 2.**
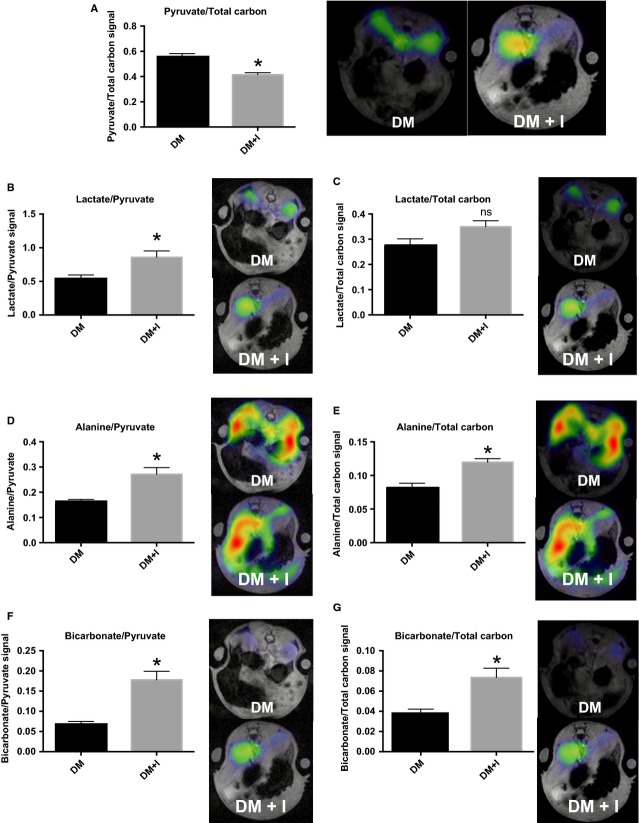
Metabolic parameters. Pyruvate‐to‐total‐carbon (A), lactate‐to‐pyruvate (B), Lactate‐to‐total‐carbon (C), alanine‐to‐pyruvate (D), Alanine‐to‐total‐carbon (E) bicarbonate‐to‐pyruvate (F) and bicarbonate‐to‐total‐carbon (G) ratios and representative images of kidneys from diabetic rats with (DM + I) and without suboptimal insulin treatments (DM). *denotes *P* < 0.05 versus untreated diabetes.

The metabolism of hyperpolarized pyruvate is illustrated in the diagram (Fig. 1), where the hyperpolarized signals are illustrated in red. The metabolic maps of lactate, alanine, pyruvate, and bicarbonate were overlaid on the anatomical ^1^H‐based images. Increased substrate utilization in the kidneys of diabetic rats receiving insulin was evident as reduced pyruvate‐to‐total carbon ratio, with a difference between the treated and the untreated diabetic rats of 0.14 ± 0.03 (*P* = 0.002; Fig. 2A). The increased pyruvate utilization in these kidneys resulted in an increased lactate‐to‐pyruvate ratio of 0.35 ± 0.13 (*P* = 0.03), alanine‐to‐pyruvate ratio of 0.12 ± 0.03 (*P* = 0.01; Fig. 2B and C) in the diabetic‐insulin‐treated (DM + I) rat kidneys compared with untreated diabetic (DM) rat kidneys, indicating increased anaerobic metabolic flux.

**Figure 3.**
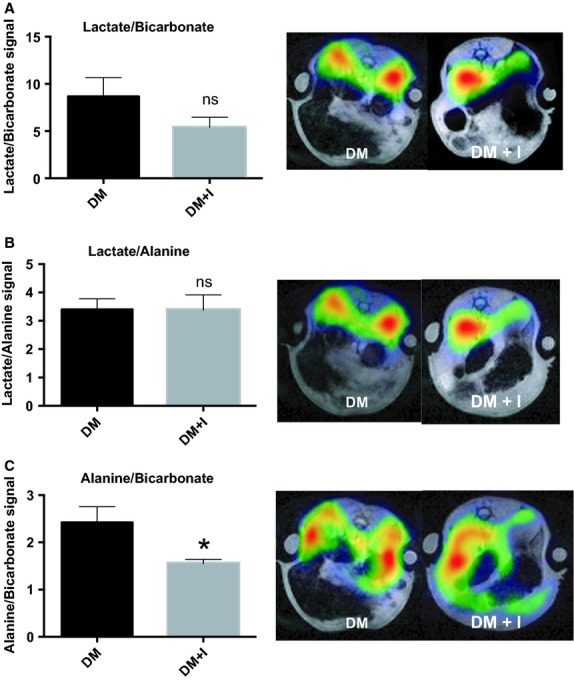
Lactate‐to‐bicarbonate (A), lactate‐to‐alanine (B), and alanine‐to‐bicarbonate (C) ratios and representative images of kidneys from diabetic rats with (DM + I) and without (DM) suboptimal insulin treatments. *denotes *P* < 0.05 versus untreated diabetes.

**Figure 4.**
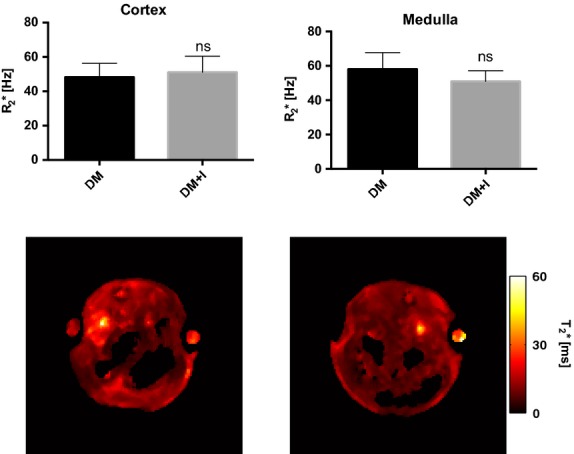
Kidney cortex and medulla R_2_* values in diabetic rats with and without suboptimal insulin treatments.

Additionally, an 0.11 ± 0.03 increase in the bicarbonate‐to‐pyruvate ratio was found in the diabetic‐insulin‐treated rat kidneys compared with untreated diabetic rat kidneys (*P* = 0.002), indicating increased aerobic metabolic flux (Fig. 2D). The balance between anaerobic and aerobic metabolism (lactate‐to‐bicarbonate ratio) was similar in the two groups (*P* = 0.16; Fig. 3A). The balance between the two anaerobic pathways investigated (lactate‐to‐alanine ratio) was similar in the two groups (*P* = 0.97; Fig. 3B), whereas the balance between the amino acid synthase and aerobic metabolism (alanine‐to‐bicarbonate ratio) was reduced by 0.87 ± 0.31 in the diabetic rats receiving insulin (DM + I) compared with untreated diabetic rats (DM; *P* = 0.02; Fig. 3C). Blood‐oxygen‐level‐dependent contrast (R_2_*) was similar in both cortex and medulla in the two groups (*P* = 0.83 and *P* = 0.54, respectively), indicating similar renal oxygenation levels in the two groups (Fig. 4).

## Discussion

The main finding of this study is that insufficient insulin administration accelerates the utilization of the energy substrate pyruvate, suggesting both increased anaerobic and aerobic metabolic fluxes. The relative increase in the two anaerobic and aerobic pathways was similar, indicating that the diabetic kidney is under general substrate‐dependent‐metabolic control.

Poor glycemic control is the main risk factor for diabetic nephropathy in both type 1 and type 2 diabetic patients (Richard et al. 2011). On the other hand, good glycemic control has been shown both clinically and experimentally to prevent the onset and to slow down the progression of already established diabetic nephropathy (Richard et al. 2011). However, a number of patients never achieve normal regulation of blood glucose levels and therefore endure with chronically increased concentrations of HbA_1c_ (The Diabetes Control and Complications Trial Research Group 1993). These patients resemble the experimental group receiving “suboptimal or insufficient” insulin treatment, which in this animal study corresponds to an accelerated metabolic alteration compared to untreated diabetic group. It should be noted that this study does not define a specific glycemic threshold above which suboptimal insulin administration affects kidney metabolism in a more negative way compared to the potentially beneficial effects achieved by the slight, but significant, reduction in blood glucose levels.

The mechanism for the observed accelerated metabolic changes induced by insufficient insulin administration in the presence of hyperglycemia may originate from the cellular uptake of glucose, which is mediated by glucose transporters (GLUTs) with distinct tissue‐specific expressions (Bell et al. 1990). The renal expression of the insulin‐responsive GLUT4 is localized to microvessels, glomeruli, mesangial cells (Brosius et al. 1992), and thick ascending limb (Edward Chin et al. 1997). Both mRNA and protein levels of GLUT4 are down‐regulated already 1 week after diabetes in rats (about 30% and 50%, respectively), resulting in a 54% decreased cellular uptake of deoxyglucose (Marcus et al. 1994). It is likely that the applied suboptimal insulin administration restored cellular glucose uptake, which should contribute to the increased metabolism observed in this study. Thus, it seems that a further deranged kidney metabolism is protected by the lack of insulin in the STZ rat model via the parallel increase in the aerobic and anaerobic pathways.

Lactate dehydrogenase and pyruvate dehydrogenase convert pyruvate to either lactate or acetyl‐CoA, respectively. An unaltered oxygen‐dependent entrance into the Krebs cycle via pyruvate dehydrogenase indicates sufficient oxygen supply to the diabetic kidney to conduct oxidative phosphorylation. Note the energy potential of anaerobic glycolysis is only 2 ATP molecules per glucose molecule, compared to oxidative phosphorylation with an energy potential of 36 ATP molecules per glucose molecule. Alanine aminotransferase facilitates the reversible conversion of pyruvate and glutamate into alanine and *α*‐ketoglutarate, which has been shown to correlate strongly with cellular oxygen availability (Laustsen et al. 2014). Hypoxia shifts glutamine metabolism from oxidation to reductive carboxylation, manifested as increased *α*‐ketoglutarate‐to‐citrate ratio (Fendt et al. 2013). The alanine pool is therefore directly regulated by glutamine metabolism and in turn the fatty acid synthesis via the cosubstrates glutamate and *α*‐ketoglutarate. Early changes in the diabetic kidney are associated with decreased pyruvate‐to‐alanine conversion, whereas the pyruvate‐to‐alanine conversion normalizes during overt hyperglycemia (Laustsen et al. 2013, 2014), indicating alanine as a sensitive marker for changes in the diabetic kidney.

It has previously been shown that hyperglycemia induces pseudohypoxia, defined as increased lactate formation even though the cellular oxygen supply is sufficient to run oxidative phosphorylation (Williamson et al. 1993). This is indicated by the unaltered lactate‐to‐bicarbonate ratio between the insulin treated rats compared to the nontreated diabetic rats in this study. We have previously reported that pseudohypoxia is accelerated as the oxygen supply decreases (Laustsen et al. 2014). The novelty of this study relates to the finding that the metabolic fluxes through these anaerobic pathways are tightly regulated by insulin, possibly by directly affecting the cellular substrate availability for the energy metabolism.

The lactate‐to‐bicarbonate ratio was unaffected by the insulin treatment, whereas reduced alanine‐to‐bicarbonate ratio was observed, indicating that it is possible to differentiate pseudohypoxia from true hypoxia or glutamine and fatty acid metabolism by invasive hyperpolarized ^13^C‐pyruvate MRI. The similar kidney R_2_* values indicate similar kidney oxygenation in both groups, suggesting that differences in oxygen levels cannot explain the observed effects of insulin on renal metabolism.

In conclusion, insulin promotes general metabolic activity, while good glycemic control alleviates or slows the progression of diabetic nephropathy, suboptimal insulin administration to insulinopenic rats with poor glycemic control accelerates the renal metabolic disturbances, including both anaerobic and aerobic pathways. The accelerated pyruvate utilization flux after insulin administration indicates that the metabolic disturbances in the diabetic kidney during poor glycemic control are limited by substrate availability, possibly indicating a novel therapeutic target to treat diabetic nephropathy.

## Acknowledgments

S. Gude and L. Nielsen are acknowledged for their expert laboratory assistance.

## Conflict of Interest

None declared.
